# Effect of the Nanotube Radius and the Volume Fraction on the Mechanical Properties of Carbon Nanotube-Reinforced Aluminum Metal Matrix Composites

**DOI:** 10.3390/molecules26133947

**Published:** 2021-06-28

**Authors:** Myung Eun Suk

**Affiliations:** Mechanical Engineering, IT Convergence College of Materials and Components Engineering, Dong-Eui University, Busan 614-714, Korea; msuk@deu.ac.kr

**Keywords:** carbon nanotube, aluminum, metal matrix composite, mechanical properties, molecular dynamics simulation, CNT-Al

## Abstract

By using the advantages of carbon nanotubes (CNTs), such as their excellent mechanical properties and low density, CNT-reinforced metal matrix composites (MMCs) are expected to overcome the limitations of conventional metal materials, i.e., their high density and low ductility. To understand the behavior of composite materials, it is necessary to observe the behavior at the molecular level and to understand the effect of various factors, such as the radius and content of CNTs. Therefore, in this study, the effect of the CNT radius and content on the mechanical properties of CNT-Al composites was observed using a series of molecular dynamics simulations, particularly focusing on MMCs with a high CNT content and large CNT diameter. The mechanical properties, such as the strength and stiffness, were increased with an increasing CNT radius. As the CNT content increased, the strength and stiffness increased; however, the fracture strain was not affected. The behavior of double-walled carbon nanotubes (DWNTs) and single-walled carbon nanotubes (SWNTs) was compared through the decomposition of the stress–strain curve and observations of the atomic stress field. The fracture strain increased significantly for SWNT-Al as the tensile force was applied in the axial direction of the armchair CNTs. In the case of DWNTs, an early failure was initiated at the inner CNTs. In addition, the change in the elastic modulus according to the CNT content was predicted using the modified rule of mixture. This study is expected to be useful for the design and development of high-performance MMCs reinforced by CNTs.

## 1. Introduction

As carbon nanotubes (CNTs) exhibit excellent mechanical properties, polymer and metal matrix composite (MMC) materials reinforced with CNTs have been actively developed. In particular, since the importance of developing lightweight materials is increasing from an environmental point of view, CNT composite materials have been studied for the purpose of supplementing the low strength of polymers or low-density metals. In the aerospace, automotive, and railway industries, the usage of lightweight materials plays an important role in saving fuel and electricity and, thus, reducing carbon emissions. Composites reinforced with CNTs in low-density metals, such as aluminum and magnesium, have been actively developed and studied in the last decade [1,2]. In addition, a low-density metal foam coated by graphene was developed to sustain the low density and high strength [3]. Recently developed CNT-reinforced carbon matrix (CNT-C) composites have also shown great potential as lightweight, strong, and highly conductive materials [4,5].

Carbon nanotube-aluminum (CNT-Al) MMCs can be produced in a variety of ways, such as powder metallurgy, thermal spraying, melt processing, etc. Among the many methods, including novel techniques, powder metallurgy [6,7] was reported as the best technique for mass production and cost-effectiveness [8]. Researchers reported a 50–113 % increase in tensile strength and 23–57 % increase in stiffness with a 3–5 % CNT content [9,10]. When fabricating composite materials reinforced by CNTs, not only the homogeneity of the nanotubes but also various factors, e.g., the content, radius, and interfacial properties of CNTs, affect the mechanical properties of the final composite materials [11,12]. Molecular dynamics (MD) simulations are a good method to systematically study the independent effects of such factors on the mechanical properties of materials and have provided atomic-scale details for mechanical behaviors of MMCs.

The compressive [13,14] and tensile behaviors [15] of CNT-Al have been studied using MD simulations. The increase in Young’s modulus was observed in accordance with the experimental study. The increase was attributed not only to the intrinsic stiffness of the CNTs but also to interfacial shear stress. The Ni coating of CNT further increased the Young’s modulus of CNT-Al MMCs by improving the interfacial bonding between Al and CNT [16]. The effects of the temperature [17], CNT radius [15], and volumetric contents [13] on the mechanical properties were studied using MD simulations.

Studies of CNT-Al MMCs where the CNTs have a large radius close to the experimental scale or high content that are advantageous as lightweight materials have been insufficient. Therefore, in this study, the influence of a wide range of nanotube radii and contents was systematically observed using MD simulations. In addition, the present study will be useful in the development of CNT-Al MMCs by presenting a predictive model of stiffness encompassing the experimental results.

## 2. Results and Discussion

### 2.1. Tensile Testing and Stress–Strain Curve

**Figure 1:**
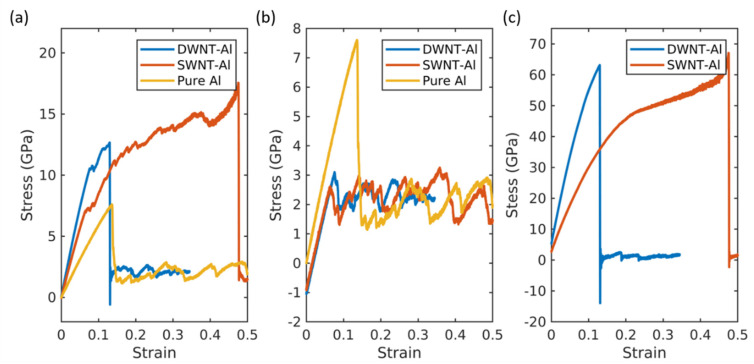
(**a**) The total stress–strain curves for the DWNT-Al composite, SWNT-Al composite, and pure aluminum. (**b**) Aluminum matrix stress–strain curves for the DWNT-Al composite, SWNT-Al composite, and pure aluminum. (**c**) CNT stress–strain curves for the DWNT-Al composite and SWNT-Al composite.

The representative stress–strain curves for single walled carbon nanotube-aluminum (SWNT-Al) and double walled carbon nanotube-aluminum (DWNT-Al) are plotted in Figure 1. The corresponding CNT wt% is approximately 10.5. The radius of the SWNTs were chosen to be similar to the inner radius of the DWNTs. The stress–strain curves for the CNT and Al matrix are plotted in Figure 1b,c, respectively, by calculating the stress components of the CNT part and matrix part separately. When Equation (11) is applied to calculate the stress, summation is performed over carbon or aluminum separately with the corresponding CNT or Al matrix volume. Therefore, Figure 1b contains the stress withstood by the aluminum and Figure 1c contains the stress withstood by SWNTs or DWNTs.

As shown in Figure 1, the CNT-Al composites had enhanced mechanical properties compared with the pure aluminum. The overall mechanical properties, e.g., the stiffness, strength, and toughness, were enhanced as summarized in Table 1. The SWNT-Al and DWNT-Al composites exhibited distinct features in their tensile behavior. The stiffness was larger for the DWNT-Al composite, while the toughness and ultimate tensile strength were larger for the SWNT-Al composite. The higher stiffness of the DWNT-Al composites was attributed to the higher stiffness of DWNTs as can be seen in Figure 1c. Despite the higher stiffness of DWNTs, the strength and ductility were higher for SWNT-Al composites compared with those of DWNT-Al composites. As can be seen in Figure 1c, the extended failure strain of the CNT contributed to the higher strength and ductility of the SWNT-Al composite. For both SWNT-Al and DWNT-Al composites, the failure occurred in the CNT reinforcements.

### 2.2. Stress Field and Fracture of Carbon Nanotubes

**Figure 2:**
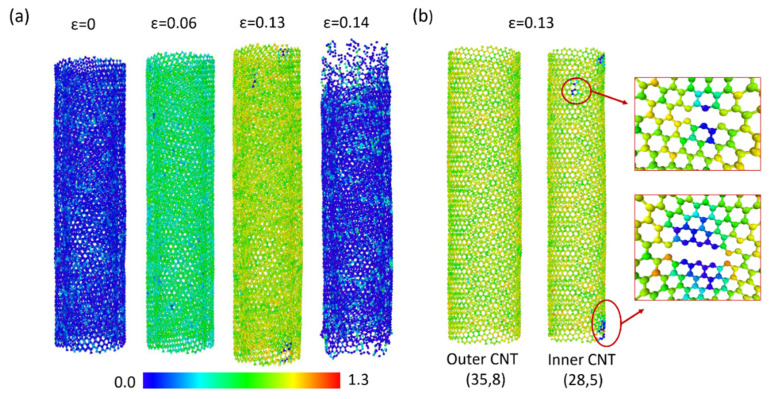
(**a**) CNT stress field changes with the strain for DWNT-Al. (**b**) Stress field and bond breakage at the fracture strain. The color bar represents the magnitude of the atomic stress in 10^3^ GPa ${Å}^{3}$.

**Figure 3:**
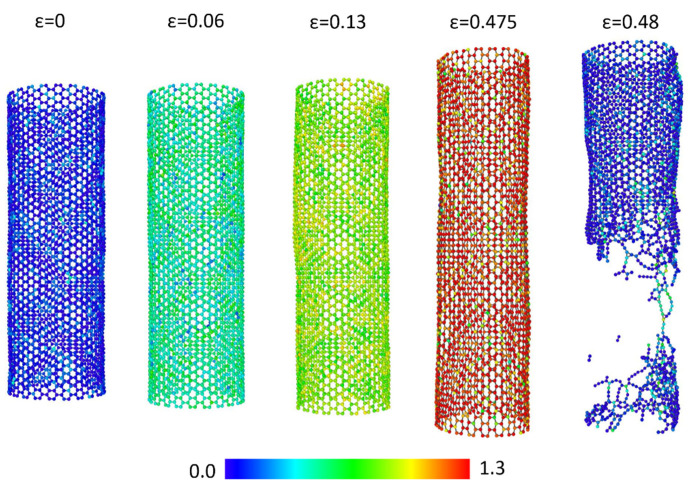
CNT stress field changes with the strain for SWNT-Al. The color bar represents the magnitude of the atomic stress in 10^3^ GPa ${Å}^{3}$.

To explain the distinct tensile behaviors of the SWNT-Al and DWNT-Al composites, the CNT stress field and failure mechanism were investigated. The stress field is obtained at the (1) aluminum matrix yield point (ε=0.06), (2) failure point of the DWNT-Al composites (ε = 0.13), and (3) failure point of the SWNT-Al composites (ε = 0.48). The stress fields for DWNTs and SWNTs are displayed in Figure 2 and Figure 3, respectively. In Figure 2a, the atomic stress calculated by Equation (11) is colored according to its magnitude, and the variations with the strain are represented.

The blue colored atoms shown for zero strain represent atoms holding near zero stress. At a strain of 0.06, the mean atomic stress was higher for the inner CNTs ($\bar{\sigma_{zz}}=557$ GPa ${Å}^{3}$) compared with the outer CNTs ($\bar{\sigma_{zz}}=524$ GPa ${Å}^{3}$). At a strain of 0.13, three regions experiencing low stress are spotted at the inner CNTs. The inner CNTs and outer CNTs are displayed separately in Figure 2b for ε=0.13. As can be seen in Figure 2b, bond breakage or low atomic stress were not observed in the outer CNTs. In the inner CNTs, a series of bond breakage forming cracks were observed, and the several associated atoms lost load bearing capacitance. At a strain of 0.14, the complete fracture of DWNTs was observed at both the inner and outer CNTs.

Figure 3 displays the atomic stress field of SWNTs during the tensile tests of SWNT-Al composites. During elastic deformation, the stress distribution of SWNT showed similar behavior with the DWNTs. Around ε=0.06, the mean atomic stress increased to 481 GPa ${Å}^{3}.$ At a strain of 0.13, bond breakage and low stress regions were not observed in SWNTs, and the mean atomic stress was increased up to 855 GPa ${Å}^{3}.$ Up to the failure strain of 0.475, SWNTs showed stable hexagonal structures with an extended C-C bond length.

The carbon atoms that underwent high stress were widely distributed. During the super linear increment of stress–strain curves, high population of highly stressed carbon atoms were observed. Around the failure strain, the sudden rupture of SWNTs was found. The ruptures were initiated by the simultaneous breakage of carbon bonds. The extended failure strain of SWNT-Al composites may be due to the loading direction coinciding with the direction of the armchair SWNTs. We observed that the arrangement of atoms was balanced with the extended C-C bond length as the strain was attained in the direction of the armchair CNTs. When testing CNT-Al composites with (35,8) SWNT, the fracture strain decreased to 0.31.

### 2.3. Effect of Volume Fraction

**Figure 4:**
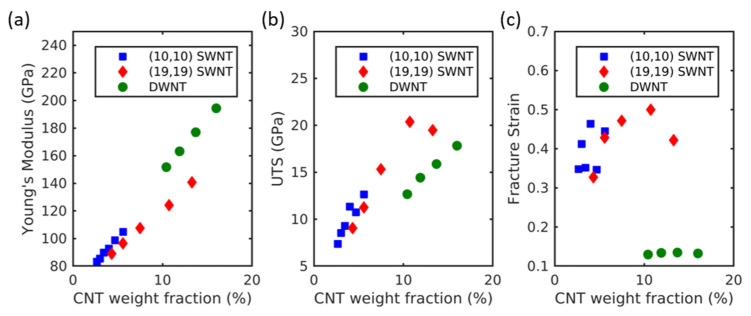
(**a**) Young’s modulus, (**b**) ultimate tensile strength, and (**c**) fracture strain of CNT-Al composites with the variation of the CNT weight fraction.

The effect of the weight percentage of CNTs on the mechanical properties was investigated by varying the simulation cell sizes. (10,10) SWNT-Al, (19,19) SWNT-Al, and (28,5)@(35,8) DWNT-Al composites were used to test various weight fractions of CNTs. The elastic moduli, ultimate tensile strength, and fracture strain are displayed in Figure 4. The elastic modulus and ultimate tensile strength increased overall with the increasing weight fractions of the CNTs. These trends were observed for both SWNT-Al and DWNT-Al composites regardless of the CNT radii. This is consistent with the rule of mixture where increasing CNT volume fractions results in increased elastic moduli. The ultimate tensile strength also increased with the increasing wt% of CNTs for both SWNT-Al and DWNT-Al composites.

The comparison of SWNT-Al and DWNT-Al composites with similar CNT wt% showed that the strength of SWNT-Al was higher than that of DWNT-Al composites, while the elastic modulus of DWNT-Al was higher than that of SWNT-Al composites. The high strength of SWNT-Al composites is due to the high fracture strain compared with that of DWNT-Al composites. As can be seen in Figure 4c, the failure strain was significantly larger for SWNT-Al composites compared with that of DWNT-Al composites. In addition, SWNT-Al composites exhibited large variations in the fracture strain, while the DWNT-Al composites exhibited a nearly constant failure strain. Despite the large variation in the failure strain of SWNT-Al composites, no general trend of variation with the CNT wt% was observed. As discussed in Section 2.2, the delayed failure strain may be a result of the stable configuration of armchair CNTs under the high mechanical strain.

### 2.4. Effect of CNT Radius

**Figure 5:**
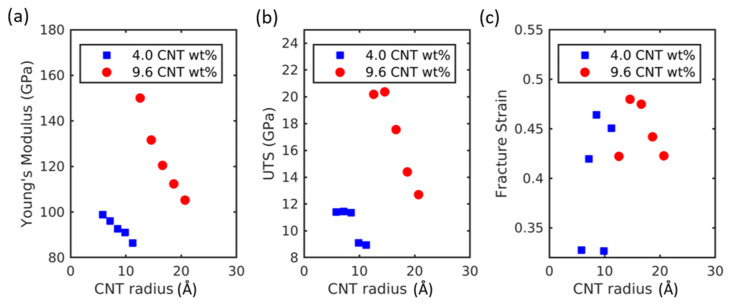
(**a**) Young’s modulus, (**b**) ultimate tensile strength, and (**c**) fracture strain of CNT-Al composites with the variation in the CNT radius.

The effect of the CNT radius on the tensional properties of CNT-Al composites was investigated. The wide ranges of CNT radii were tested by inserting (6,6)–(28,28) SWNTs. The weight fraction of CNTs was kept constant at 4.0 and 9.6 wt%. The Young’s modulus decreased with the increasing radius of CNT. The difference of the measured Young’s modulus was about 12 GPa, while the radius was changed by 5.6 nm for the 4.0 CNT wt%. This was about 45 GPa, while the radius was changed by 8 nm for the 9.6 CNT wt%. The higher CNT contents indicate that the influence of CNT radius was larger. The ultimate tensile strength (UTS) also decreased with the increasing CNT radius. The changes in UTS include statistical variations due to the effect of the fracture strain. As can be seen in Figure 5c, the fracture strain includes statistical variations irrelevant to the CNT radius.

The circular geometry of CNT may be deformed during an experimental fabrication process such as ball milling. In such a case, the extreme collapsing of circular cross-sections would approach the platelet geometry similar to graphene platelets. Sharma et al. [18] used molecular dynamics simulations to compare the mechanical properties of graphene-reinforced MMCs and CNT-reinforced MMCs. They observed that the Young’s modulus of graphene-reinforced MMCs was slightly higher than that of CNT-reinforced MMCs when the same volume contents were compared. Furthermore, higher yield strength was observed for graphene–Al compared to CNT–Al in the experimental study [19]. Thus, the collapsed circular cross-section is expected to increase the mechanical strength of MMCs at the expense of increasing density. However, the defect formation during deformation may degrade the mechanical properties of MMCs [20]. The effect of CNT deformation during the fabrication process needs to be further studied both theoretically and experimentally. As a future direction, it will be worthwhile to study the effect of geometric CNT deformation on mechanical properties by using molecular dynamics simulations.

### 2.5. Young’s Modulus

The rule of thumb relation for Young’s modulus with the CNT volume fraction is given by the two-phase model. According to the Voigt and Reuss models,

(1) $$E_{ROM}=E_{CNT}v_{CNT}+E_{Al}\left(1-v_{CNT}\right)\;$$

where $E_{CNT}$ is the Young’s modulus of the CNT, $E_{Al}$ is the Young’s of Al and $v_{CNT}$ is the volume fraction of the CNT. 

From the stress field in the matrix phase, there exists an interphase region where the stress field around the CNT is higher [13]. Therefore, the rule of mixtures can be modified by introducing the Young’s modulus of the interphase region. The modified rule of mixtures (MROM) can be given by:

(2) $$E_{MROM}=E_{CNT}^{*}v_{CNT}+E_{I}v_{I}+E_{Al}\left(1-v_{CNT}-v_{I}\right)$$

where $E_{I}$ is the Young’s modulus of the interphase region, $v_{I}$ is the volume fraction of the interphase region, and $E_{CNT}^{*}$ is the effective Young’s modulus of CNT inserted into the matrix phase.

**Figure 6:**
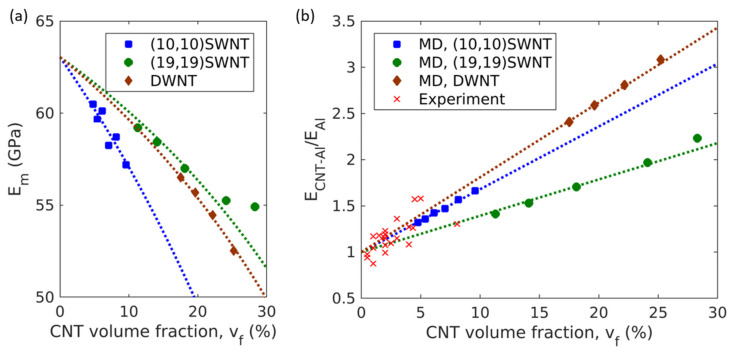
(**a**) The Young’s modulus of the matrix phase that composes CNT-Al composites. Dashed lines represent Equation (5). (**b**) The variation of the Young’s moduli of CNT-Al composites with CNT volume fractions. Dashed lines represent Equation (6).

The Young’s modulus of the interphase region can be obtained from the changes in Young’s modulus of Al matrix with the CNT volume fraction as shown in Figure 6a. The original rule of mixtures assumes that the Young’s modulus of the matrix does not depend on the CNT volume fraction. The decomposed stress–strain curves do, however, indicate that the Young’s modulus of the matrix depends on the CNT volume fraction, possibly due to the changes in the volume fraction of the interphase region. Applying the rule of mixtures for the matrix, Young’s modulus of the matrix phase is given by

(3) $$E_{m}=E_{I}\frac{V_{I}}{V_{tot}-V_{CNT}}+E_{Al}\frac{V_{tot}-V_{CNT}-V_{I}}{V_{tot}-V_{CNT}}$$

where $V_{I}$ is the volume of the interphase region,$\;V_{CNT}$ is the volume of the CNT, $V_{tot}$ is the total volume.

For the annular interphase geometry [13] embedded in square representative volume elements (RVE), the volume of the interphase region is as follows:

(4) $$V_{I}=\left(\eta^{2}+2\eta \right)V_{f}$$

where η is the ratio of the thickness of the interphase area to the CNT radius, η=d/R. Therefore, the Young’s modulus of the matrix phase can be expressed as a function of the CNT volume fraction as follows:

(5) $$E_{m}=\frac{\left\{\left(\eta^{2}+2\eta \right)\left(E_{I}-E_{Al}\right)-E_{Al}\right\}v_{CNT}+E_{Al}}{1-v_{CNT}}$$

where $v_{CNT}$ is the CNT volume fraction, and $v_{CNT}=\frac{V_{CNT}}{V_{tot}}\;$. For (10,10) SWNT, *R* can be defined as the CNT radius including the carbon radius, which is 0.848 nm. The thickness of the interphase area can be defined as *d* = 3$\sigma_{Al}=9.05$. Therefore, η=1.067, 0.621, and 0.526 for (10,10) SWNTs, (19,19) SWNTs, and DWNTs, respectively.

By fitting the Young’s modulus of matrix ($E_{m}$) vs. the CNT volume fraction ($v_{CNT}$) to Equation (5), the Young’s modulus of interphase ($E_{I})$ can be obtained. The young’s modulus obtained from the pure Al simulation was 62.22 GPa, and it was substituted for *E_Al_* in Equation (5). As can be seen in Figure 6a, the variation of $E_{m}$ with respect to the $v_{CNT}$ is well-described by Equation (5) and is represented by the dashed lines. The $E_{I}$ was determined as 46.59 GPa for both (10,10) SWNTs and (19,19) SWNTs, and it was determined as 37.97 GPa for DWNTs.

By substituting Equation (4) into Equation (2), the Young’s modulus of CNT-Al composites can be expressed as a function of the CNT volume fraction as follows:

(6) $$\;E_{CNT-Al}=\left\{E_{CNT}^{*}-E_{Al}+\left(E_{I}-E_{Al}\right)\left(\eta^{2}+2\eta \right)\right\}v_{CNT}+E_{Al}$$

The Young’s moduli of CNTs within composites were also obtained from the decomposed stress–strain curves for CNTs. They were 545.47, 337.64, and 606.89 GPa, for (10,10) SWNTs, (19,19) SWNTs, and DWNTs, respectively. As shown in Figure 6b, the modified rule of mixture expressed by Equation (6) successfully describes the variation of the composite Young’s modulus as a function of the CNT volume fraction.

In addition, the experimental data [9,21,22,23,24,25] were compared with the MD data and the MROM in Figure 6b. As the MROM was obtained for loading in the CNT axial direction, the comparison considered the possibility of CNT alignments during the extrusion in the experimental process. Due to the dispersion problems observed in experiments, the experimental data are shown for the CNT volume fractions up to 10%. The experimental data exhibited large variance as the fabrication process and CNT distribution affected the mechanical properties; however, the experimental data are in general agreement with the MD data and the MROM equation.

## 3. Materials and Methods

### 3.1. Simulation Set-Up

**Figure 7:**
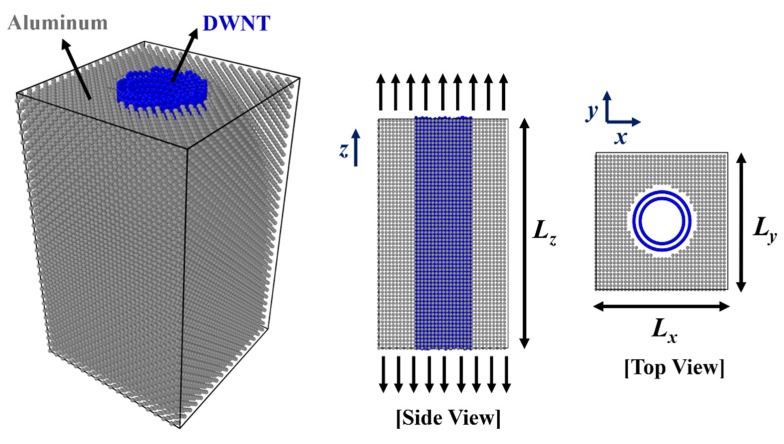
CNT-Al composite RVE for the MD simulations. *L_z_* =124.44 Å and *L_z_*=97.25 Å for DWNT-Al and SWNT-Al, respectively. *L_x_* and *L_y_* were adjusted from 44.5 to 89.1 Å according to the CNT volume fraction and CNT size.

The RVE for MD simulations were composed of the vertically aligned CNTs in the Al matrix as shown in Figure 7. In simulating DWNT-Al MMCs, experimentally observed (28,5)@(35,8) DWNTs [26] were used as reinforcements. Experiments have shown that DWNTs in AA stacking were unstable [26]. DWNTs with zero helicities difference (Δθ = 0) such as (n,n)@(m,m) types or (n,0)@(m,0) types were not observed in experiments. The approximate helicities difference (Δθ) of (28,5)@(35,8) DWNT was 2.0°, and the inner and outer tube radii were 12.06 and 15.51 Å, respectively. In simulations of SWNT-Al MMCs, (6,6), (8,8), (10,10), (12,12), (14,14), (16,16), (19,19), (22,22), (25,25), and (28,28) SWNTs were inserted in the Al matrix. The corresponding SWNT radii were 4.06–18.98 Å.

To utilize the periodic boundary conditions, the size of the RVE should be the multiplication of the lattice constants of Al, which is 4.05 Å. In addition, the height of the RVE should be coincident with the super cell sizes of CNT. By thoroughly investigating the possible multiplications of the lattice constant, coincident super cell sizes with 2% error were selected for the height of the RVEs. They were 97.25 and 124.44 Å for SWNT-Al and DWNT-Al, respectively. The lateral size of RVE was determined to produce the target CNT volume fraction, which is in the range of 4.7–28.3 vol%. The simulation cell contained 9468–39,865 atoms.

### 3.2. Interatomic Potential

In the MD simulations, the forces acting on individual atoms were calculated from the interatomic potentials, which have been developed in various forms to accurately describe atomic interactions. Lennard–Jones (LJ) potentials have been widely adopted to represent van der Waals interactions. To describe carbon and aluminum van der Waals interactions, the LJ potential was implemented according to Equation (7).

(7) $$V_{LJ}=\frac{1}{2}\sum_{i}\sum_{j\neq i}4\varepsilon_{ij}\left[{\left(\frac{\sigma_{ij}}{r_{ij}}\right)}^{12}-{\left(\frac{\sigma_{ij}}{r_{ij}}\right)}^{6}\right]$$

where $\varepsilon_{ij}$ and $\sigma_{ij}$ are the LJ energy and distance parameters, respectively. The parameters for the aluminum and carbon interactions were calculated using the Lorentz–Berthelot mixing rule where $\sigma_{ij}=\frac{\sigma_{ii}+\sigma_{jj}}{2}$ and $\varepsilon_{ij}=\sqrt{\varepsilon_{ii}\varepsilon_{jj}}$. With the carbon parameters [27] and aluminum parameters [28], $\sigma_{Al-C}=3.0135\;Å$ and $\varepsilon_{Al-C}=0.03508\;\mathrm{eV}$.

To describe the carbon interactions in CNTs, the adaptive intermolecular reactive empirical bond order (AIREBO) potential was used. In the AIREBO potential, the REBO potential is modified to include non-bonded van der Waals interactions and torsional interactions. The van der Waals interactions are represented with the LJ potential as in Equation (7). The AIREBO potential can be summarized via Equation (8) [29].

(8) $$V_{AIREBO}=\frac{1}{2}\sum_{i}\sum_{j\neq i}\left[V_{ij}^{REBO}+V_{ij}^{LJ}+\sum_{k\neq i,j}\sum_{l\neq i,j,k}V_{kijl}^{TORSION}\right]$$

where $V_{ij}^{REBO}$ represents the covalent bonding interactions of atoms based on the second-generation reactive empirical bond order potentials of Brenner, $V_{ij}^{LJ}$ is the LJ potential, and $V_{kijl}^{TORSION}$ is the torsional interactions depending on the dihedral angle of the system.

Metallic interactions have been successfully described with the embedded atom method (EAM) potential. The EAM potential is represented by:

(9) $$V_{EAM}=\sum_{i}F_{i}\left(\rho_{i}\right)+\frac{1}{2}\sum_{i}\sum_{j\neq i}\phi \left(r_{ij}\right)$$

where F(ρ) is the embedding energy as a function of the local electron density ρ, and $\phi \left(r_{ij}\right)$ is the pairwise interaction as a function of the distance between two particles. The local electron density induced at site *i* is calculated by the atomic density function of all other atoms.

(10) $${\bar{\rho }}_{i}=\sum_{j\neq i}\rho \left(r_{ij}\right)$$

The EAM potential functions and parameters for Al have been reported by many authors. This study adopted the parameters developed by Mishin et al. [30], as the mechanical properties of Al are close to the experimentally obtained values. The MD results were in good agreement with the experimental results regarding the elastic constants, surface energies, migration energies, stacking fault energies, and vacancy formation [30]. Notably, the elastic modulus obtained from the MD was 61.43 GPa [13], which is in close agreement with the experimentally obtained elastic modulus of 60.2–69.5 GPa [31,32].

### 3.3. Simulation Procedure

As the first step, energy minimizations and equilibrium simulations were performed to stabilize the carbon and Al interface. The equation of motion was integrated with a time step of 0.5 fs. To attain the NPT ensemble, the temperature and pressure were maintained at 300 K and 0 bar, respectively, using the Nosé–Hoover algorithm. After equilibration for 100 ps, the mechanical strain was applied in the *z*-direction with a constant strain rate of $10{\;\mathrm{ns}}^{-1}$. Tensile simulations were performed until the fracture occurred. The stress was calculated using the virial theorem, which is calculated by

(11) $$\sigma \left(r^{N}\right)=\frac{1}{V}\left[\sum_{i=1}^{N}m_{i}v_{i}⊗v_{i}+\sum_{n\in Z^{3}}^{}\sum_{i=1}^{N}r_{in}⊗F_{in}^{\prime }\right]$$

where *i* and *N* are the atom index and total number of atoms, respectively, in the local cell, $n\in Z^{3}$ is a vector of three integers representing the *x*, *y*, and *z* offsets of the periodic images relative to the local cell, *m_i_* and *v_i_* are mass and velocity of atom *i*, respectively, $r_{in}$ is the position vector of atom *i*, and $F_{in}^{\prime }$ is the partial force on atom *i* due to the local cell.

## 4. Conclusions

In this study, the tensile behavior of aluminum metal matrix composites with large CNT radii and contents were observed using molecular dynamics simulations. Through tensile simulations and decomposition of the stress–strain curve, it was confirmed that the increased strength of the composites was due to the high strength of CNTs. In particular, DWNTs showed greater elastic moduli compared to those of SWNTs; however, the fracture strain was smaller for DWNTs compared to SWNTs. In the case of the fracture strain, there was no significant difference between a pristine aluminum material and DWNT-Al composite; however, this was highly increased for the SWNT-Al composites.

From the stress field investigation and atomic arrangement of CNTs, it was estimated that the tensile force applied in the armchair CNT direction contributed to the increased ductility of the SWNT-Al composites. In the investigation of the effect of the CNT radius, the strength of the CNT-Al composites increased with the decreasing CNT radius. As the CNT content increased, the stiffness and strength of CNT-Al increased, but the fracture strain was not significantly affected. To predict the change in Young’s modulus according to the CNT content, a modified rule of mixture was proposed considering the interphase area of the Al matrix adjacent to the CNTs. The experimental data and MD data were found to be in reasonable agreement with the modified rule of mixtures.

## Figures and Tables

**Table 1:** Mechanical properties obtained from the stress–strain curves.

	Inner Radius (Å)	wt%	Young’s Modulus (GPa)			Tensile Strength (GPa)	Fracture Strain	Fracture Toughness (GPa)
			CNT-Al MMC	Al-Matrix	CNT			
Pure Al	-	-		62.22		7.59	0.14	0.57
DWNT-Al	17.21	10.4	151.80	56.51	600.94	12.64	0.13	1.05
SWNT-Al	16.6	9.14	120.49	56.83	327.61	17.54	0.48	5.57

## Data Availability

The data presented in this study are available in article.
